# Relationship of Growth Differentiation Factor-15, E-Cadherin, Galectin-3, and Psychological Stress with Quality of Life and Prostate Size in Benign Prostatic Hyperplasia

**DOI:** 10.5152/tud.2025.24189

**Published:** 2025-12-05

**Authors:** Palani Balamurali, Hanumanthappa Nandeesha, Sreerag K Sreenivasan

**Affiliations:** Department of Biochemistry and Urology, JIPMER, Puducherry, India

**Keywords:** Benign prostatic hyperplasia, growth differentiation factor, inflammation, perceived stress

## Abstract

**Objective:**

Inflammation and oxidative stress are involved in the pathogenesis of benign prostatic hyperplasia (BPH). The relationship of psychological stress, oxidative stress, and inflammation with prostate enlargement is unclear. The objective of the study was to investigate the relationship of growth differentiation factor-15 (GDF-15), E-cadherin, galectin-3 and psychological stress with quality of life (QOL) and prostate size in BPH patients.

**Methods:**

Eighty-two BPH cases were included in the study. Growth differentiation factor-15, galectin-3, and E-cadherin were analyzed in all the subjects. Perceived Stress Scale-10 (PSS-10) was used to analyse psychological stress. Quality of life score due to urinary symptoms was based on a single question recommended by the International Consensus Committee, and its answer range from delighted (score-0) to terrible (score-6)

**Results:**

E-cadherin (*P* = .014), galectin-3 (*P* = .004), QOL score (*P* = .024) and PSS-10 score (*P* = .001) were higher in BPH cases with increased prostate size. Growth differentiation factor-15 was lower in BPH patients with higher PSS-10 score (*P* = .036) and higher QOL score (*P* = .016). Growth differentiation factor-15 was negatively associated with International Prostate Symptom Score (IPSS) score (*r* = −0.314, *P* = .004), QOL (*r* = −0.284, *P* = .009), and PSS-10 score (*r* = −0.031, *P* = .001). Prostate size was positively associated with E-cadherin (*r* = 0.317, *P* = .003), galectin-3 (*r* = 0.243, *P* = .026), IPSS (*r* = 0.301, *P* = .005) and QOL score (*r* = 0.237, *P* = .030). Also, a significant correlation was observed between E-cadherin and galectin-3 (*r* = 0.292, *P* = .007). Growth differentiation factor-15 (*P* = .018) and E-cadherin (*P* = .029) were found to predict QOL and E-cadherin (*P* = .013) was found to predict prostate size in BPH.

**Conclusion:**

E-cadherin can predict prostate enlargement and along with GDF-15, can predict QOL in BPH.

**Main Points**

- E-cadherin, galectin-3 and Perceived Stress Scale-10 (PSS-10) score were enhanced in BPH cases with prostate enlargement.
- Growth differentiation factor-15 was negatively related to quality of life (QOL) score and PSS-10 score.
- Prostate size was positively associated with E-cadherin, galectin, International Prostate Symptom Score, and QOL score.

## Introduction

Benign prostatic hyperplasia (BPH), a prevalent disorder among men with advanced age, significantly impacts the quality of life (QOL).^1^ Earlier researchers have documented an inverse relation between BPH symptoms and QOL and concluded that BPH affects QOL by inducing psychological stress.^2^ Psychological stress was reported to be involved in the development of tumors through alteration of endocrine mechanisms.^3^ Earlier studies have found that stress was associated with BPH.^4^ Inflammation is known to be involved in prostate enlargement.^5^ The relationship of psychological stress and inflammation with prostate enlargement is still unclear.

Growth differentiation factor-15 (GDF-15), a cytokine, is expressed in several cells including macrophages and endothelial cells.^6^ Stress conditions are reported to induce GDF-15, and elevated levels are documented in inflammatory conditions like myocardial infarction and cancer.^7^ Growth differentiation factor-15 has been linked to the development and progression of tumors, clinical outcomes, and treatment response.^8^ Growth differentiation factor-15 expression has been demonstrated in premalignant and malignant conditions of the prostate.^9^

Galectin-3 is a cell adhesion molecule (CAM) involved in inflammation and regulation of apoptosis.^10^ Galectin-3 is considered a biomarker for the diagnosis and prognosis of renal and cardiac disorders and cancer.^11^ Galectin-3 expression was considered to be useful in predicting biochemical recurrence of tumors.^12^ Previous investigators have documented contradictory findings about galectin-3 expression in prostate tumors. Knapp et al,^13^ have reported a reduction in expression in tumor tissue compared to benign tissue, whereas Gao et al^14^ have demonstrated overexpression of galectin-3 in prostate cancer.

E‐cadherin is an adherens junction protein involved in the development and maintenance of tight junctions. Alteration in E‐cadherin expression results in defective tight junctions, leading to epithelium barrier dysfunction and an increase in permeability.^15^^,^^16^ E‐cadherin expression was demonstrated to be downregulated in BPH specimens compared to normal prostate tissues by earlier investigators.^17^^,^^18^

The aim of the study was to analyze the relation of growth differentiation factor-15 and psychological stress with prostate size, galectin-3, E-cadherin, and QOL in BPH.

## Materials and Methods

The current study was done in a tertiary care hospital, India from February 2023 to March 2024, and the Ethics Committee of the institute (IEC/2022/022) gave permission to conduct the study. All the subjects were informed and obtained written consent regarding the study. Eighty-four BPH patients (age = 50-75 years) admitted with lower urinary tract symptoms were enrolled in the study based on inclusion and exclusion criteria.

### Inclusion Criteria

Benign prostatic hyperplasia was diagnosed on the basis of clinical findings, per rectal examination, ultrasound findings, and followed by histopathological findings after surgery (transurethral resection of prostate). All the patients were treated with tamsulosin 0.4 mg hs for 6 months. If the symptoms were not reduced they were posted for surgery

### Exclusion Criteria

Subjects with prostate cancer, other causes of lower urinary tract symptoms, diabetes, renal disease, cardiovascular disease, and inflammatory disorders like rheumatoid arthritis, SLE, etc. were excluded from the study. Also, subjects who were on anti-inflammatory medications for a longer duration were excluded.

Prostate size was measured by ultrasound. All the patients underwent transurethral resection of the prostate, and histopathological examination of the prostate tissue was done to confirm BPH. All the patients were treated with tamsulosin 0.4 mg hs for 6 months. If the symptoms were not reduced they were posted for surgery. There are no previous studies in the literature about the effect of tamsulosin on GDF-15, galectin-3, and E-cadherin levels in BPH. Patients with medical disorders and cancer of the prostate were excluded. International Prostate Symptom Score (IPSS) was used to calculate the severity of BPH symptoms. Quality of life score due to urinary symptoms was based on a single question recommended by the International Consensus Committee, and its answer range from delighted (score-0) to terrible (score-6). Perceived Stress Scale-10 (PSS-10) was used to analyse psychological stress. Subgroup analysis was done in BPH based on the size of the prostate, QOL, and PSS-10 score.

### Sample Size Calculation

The sample size was estimated using the statistical formula for comparing 2 means with equal variance. The expected mean difference in growth differentiation factor-15 levels in BPH patients with prostate size less than 30 g and more than 30 g was 251 ng/mL.^19^ The sample size was estimated using this as the minimum expected difference at the 5% level of significance and 90% power.

### Estimation of Biochemical Parameters

Fasting blood sample (5 mL) was collected from the BPH cases and serum was separated and used for the analysis of biochemical parameters. Serum growth differentiation factor-15, galectin-3 (Krishgen Biosystems, INDIA) and E-cadherin (Elabscience, USA) were estimated by Enzyme Linked Immunosorbant Assay (ELISA).

### Statistical Analysis

The continuous data such as age GDF-15, Galectin-3, E-cadherin, prostate-specific antigen, IPSS, QOL, and PSS-10 scores were expressed as mean with SD or median (Interquartile range). Mann–Whitney *U*-test and independent student *t*-test were used to assess the differences between clinical and biochemical parameters in BPH based on prostate size (≤30 mL and >30 mL), QOL score (≤3 or >3), PSS-10 score (≤13 or >13). The association between GDF-15, galectin-3, E-cadherin, IPSS, and PSS-10 with each other and prostate size was assessed using Spearman rank correlation analysis. Multivariate regression analysis was done on the prostate size and quality of life score using age, body mass index (BMI), duration of the disease, GDF-15, galectin-3, and E-cadherin as covariates. For all statistical tests, a *P* < .05 was considered statistically significant. However, for multiple pairwise comparisons, the *P-*value was adjusted using the Bonferroni correction.

## Results

Benign prostatic hyperplasia patients presented with several clinical symptoms like incomplete emptying (20%, n = 16), frequency of less than 2 hours (25%, n = 20), intermittency (34%, n = 28), urgency (36%, n = 29), weak stream (29%, n = 32), and straining (33%, n = 27) that were present 3 or more times during/after urination. The median IPSS was 15. Among BPH cases, it was observed that 6% had mild disease (n = 5, IPSS: 1-7), 71% had moderate disease (n = 58, IPSS: 8-19), and 23% had severe disease (n = 19, IPSS: 20-35). International Prostate Symptom Score was associated with the duration of disease (*r* = 0.214, *P* = .045).

Table 1 shows the clinical characteristics, GDF-15, galectin-3, and E-cadherin in BPH participants with lower prostate size (≤30 mL) and higher prostate size (>30 mL).International Prostate Symptom Score score (*P* = .014), QOL (*P* = .024), PSS-10 score (*P* = .001), galectin-3 (*P* = .004) and E-cadherin (*P* = .014) were increased in BPH cases with increase in the size of the prostate. After applying Bonferroni correction, only galectin-3 and PSS-10 score were significant.

The biochemical and clinical characteristics in BPH cases with low stress (PSS-10 ≤ 13) and moderate/high stress (PSS-10 > 13) are shown in Table 2. Nocturia (*P* = .020), IPSS (*P* < .001), and QOL score (*P* < .001) were higher and GDF-15 (*P* = .036) was lower in BPH cases with increased PSS-10 score. However, GDF-15 and nocturia were not significant, after applying Bonferroni correction.

Table 3 shows the clinical characteristics and biochemical parameters in BPH cases with different QOL scores (≤3 and >3). Disease onset age (*P* = .042), nocturia (*P* = .001), IPSS (*P* < .001), and PSS-10 (*P* < .001) were increased and GDF-15 (*P* = .016) was decreased in the BPH cases with increase in QOL scores. However, GDF-15 was not significant, after applying Bonferroni correction.

The correlation of PSS score and GDF-15 with clinical and biochemical parameters in BPH are shown in Table 4. The PSS-10 score was negatively associated with GDF-15 (*P* = .001) and positively associated with IPSS (*P* = .001), QOL (*P* = .001), and prostate size (*P* = .001). Growth differentiation factor-15 was negatively associated with IPSS (*P* = .004) and quality of life score (*P* = .009) in BPH.

E-cadherin (*P* = .003), galectin-3 (*P* = .026), IPSS (*P* = .005), and QOL (*P* = .030) were correlated with the size of the prostate. Also, E-cadherin was correlated with galectin-3 (*P* = .007), and the QOL score was related to the nocturia score (*P* < .001) and IPSS (*P* < .001).

Multivariate linear regression analysis was performed to predict the prostate size and QOL score in BPH using age, BMI, duration of the disease, GDF-15, galectin-3, and E-cadherin as covariates (Tables 5 and 6). Growth differentiation factor-15 (*P* = .018) and E-cadherin (*P* = .029) were found to predict QOL score and E-cadherin (*P* = .013) was found to predict prostate size in BPH.

## Discussion

Benign prostatic hyperplasia patients commonly present with nocturia. In the present study, around 6% (n = 5) reported nocturia once, 13% (n = 11) reported twice, 22% (n = 17) reported 3 times, 41% (n = 34) reported 4 times, and 18% (n = 15) reported 5 times. A significant association was found between nocturia score and disease duration (*P* = .041) and QOL score (*P* = .001). Quality of life in BPH patients is known to be altered due to urinary symptoms. In the present study, around 17% (n = 14) reported that QOL was satisfactory, 68% (n = 56) were unhappy about the QOL, and 15% (n = 12) felt that QOL was terrible. Also, it was found that QOL score was positively related to prostate size (*P* = .007), suggesting prostate enlargement may result in worsening of QOL in patients with BPH.

In the current study, galectin-3, E-cadherin, and psychological stress were higher in BPH cases with a larger size of the prostate. Higher psychological stress in BPH was associated with reduced GDF-15 levels and poor QOL.

Inflammation is reported to be involved in the enlargement of the prostate and progression of BPH. Inflammatory cytokines are known to be upregulated in patients with BPH.^20^ Growth differentiation factor-15 is an anti-inflammatory cytokine, reported to be involved in the pathogenesis of BPH. Kakehi et al^21^ have documented that GDF-15 is regulated by androgens and its downregulation might play a role in the development of BPH. Taoka et al^22^ have demonstrated that GDF-15 downregulation was associated with prostatic inflammation in BPH. Bansal et al^19^ have reported that GDF-15 can be used as a marker to differentiate BPH from prostate carcinoma. In this study, GDF-15 levels were decreased in BPH cases with increase in the size of the prostate, but it was not significant (*P* = .214). Also, GDF-15 was not significantly related to prostate size (*P* = .627). When QOL due to urinary symptoms was assessed, it was found that GDF-15 was reduced in BPH cases with increased score compared to those with a lesser score (*P* = .016). These results suggest that inflammation may be associated with prostate enlargement leading to poor QOL in BPH. It was speculated that GDF-15 may cause prostate enlargement by activating signaling pathways like ERK1/2, PI3K/mTOR, MAP kinases, phosphorylation of p38, Akt and upregulating vascular endothelial growth factor.^19^ Growth differentiation factor-15 also suppresses apoptosis and promotes angiogenesis which may increase prostate size.^23^

Galectin-3 is a CAM involved in cell growth, angiogenesis, inflammation, and tumor progression.^24^ An increase in galectin-3 expression and plasma galectin-3 levels was reported in several cancers by earlier investigators.^25^ Previous studies have documented enhanced serum galectin-3 levels and its association with prostate-specific antigen in prostate cancer.^26^ Galectin-3 expression was demonstrated to increase in prostate tissues of BPH compared to normal tissue samples.^27^ In the present study, an increase in galectin-3 level was observed with an increase in prostate size in BPH (*P* = .004), indicating galectin-3 is related to enlargement of the prostate. Galectin-3 may cause prostate enlargement by increasing cell growth, adhesion, proliferation, and inhibiting apoptosis.^28^ Galectin-3 was not significantly related to prostate size in BPH patients.

E-cadherin, expressed by prostate epithelial cells, is involved in the development of prostate epithelium.^29^ Earlier researchers have documented that alteration in E-cadherin levels plays a role in the progression of prostate disease by increasing inflammation.^30^ In the present study, E-cadherin (*P* = .014) was higher in BPH cases with increase in size of the prostate and positively associated with the size of the prostate (*P* = .003) and galectin-3 (*P* = .007), suggesting E-cadherin may cause prostate enlargement through galectin-3. Multivariate analysis showed that E-cadherin (*P* = .013) predicts prostate size in BPH. E-cadherin may cause prostate enlargement through other mechanisms by altering cell-cell adhesion, increasing the permeability of prostate epithelial cells, and infiltration of inflammatory cells.^31^

Experimental studies have documented a link between stress and expression of prostate cancer related genes.^32^ Psychological stress was reported to be involved in the etiopathogenesis of BPH.^4^ In the present study, around 29% (n = 24) reported low stress (PSS-10 score: 0-13), 62% (n = 51) reported moderate stress (PSS-10 score: 14-26) and 8% (n = 7) reported high stress (PSS-10 score: 27-40) due to BPH. When subgroup analysis was done based on PSS-10 score ≤13 and >13, it was found that nocturia score (*P* = .020), IPSS (*P* < .001) and QOL score (*P* < .001) was increased and GDF-15 (*P* = .036) was reduced in BPH cases with increase in stress. Also, PSS-10 score was positively associated with prostate size (*P* = .001), IPSS (*P* = .001) and QOL score (*P* = .001) and inversely related to GDF-15 (*P* = .016). Multivariate analysis showed that GDF-15 (*P* = .018) and E-cadherin (*P* = .029) predict QOL score in BPH. These results suggest high stress is associated with prostate enlargement, inflammation, and poor QOL in BPH.

The main limitation of this study was the non-inclusion of a control group due to financial constraints. The absence of a healthy control group makes it difficult to understand whether the biomarkers are disease-specific or due to age-related changes. Most of the BPH participants were on treatment. The influence of drugs on biochemical parameters was not investigated. Postoperative stress indicators were not measured due to ethical constraints. In the present study, all the patients were non-diabetic and BMI matched. The components of metabolic syndrome were not checked due to time and financial constraints.

Based on these findings, it was concluded that galectin-3, E-cadherin, and PSS-10 score were increased and positively associated with the size of the prostate in BPH with prostate enlargement. GDF-15 was decreased in BPH with an increase in psychological stress and associated with poor QOL scores. E-cadherin was found to predict prostate enlargement and along with GDF-15, predicts QOL in BPH. The results need validation with further longitudinal molecular studies including the signaling pathways mediated by GDF-15, galectin-3, and E-cadherin.

## Figures and Tables

**Table 1.** Clinical Characteristics, Growth Differentiation Factor-15, Galectin-3, and E-Cadherin in Benign Prostatic Hyperplasia Participants with Lower Prostate Size (≤30 mL) and Higher Prostate Size (>30 mL)

Parameters	BPH with Prostate Size ≤ 30 mL (n = 42)	BPH with Prostate Size > 30 mL (n = 42)	*P*
Age (years)^a^	62 (56-66)	67 (63-72)	.004
BMI (kg/m^2^)^b^	24.0 ± 3.50	24.0 ± 3.49	.983
Nocturia (no. of times)^a^	4 (2-4)	4 (3-4)	.103
PSA (µg/L)^a^	1.31(0.49-2.45)	0.78 (0.55-0.27)	.684
IPSS^a^	14 (9-18)	16 (12-20)	.014
Quality of life^a^	3 (2-5)	4 (3-5)	.024
PSS-10 score^b^	14 ± 5.6	19 ± 5.8	<.001
GDF-15 (ng/L)^a^	1794.22 (1457.34-2478.14)	1564.55 (1058.01-2393.71)	.214
Galectin-3 (µg/L)^b^	2.41 ± 0.68	2.93 ± 0.89	.004
E-cadherin (µg/L)^a^	10.61 (5.30-51.65)	30.47 (14.25-69.16)	.014

*P*-value < .005 was considered statistically significant after adjusting for multiple comparisons using Bonferroni correction.

BMI, body mass index; BPH, benign prostatic hyperplasia; GDF-15, growth differentiation factor-15; IPSS, International Prostate Symptom Score; PSA, prostate-specific antigen; PSS-10, Perceived Stress Scale-10.

^a^Results are represented and median (range).

^b^Results are represented as mean ± SD.

**Table 2.** Clinical Characteristics, Growth Differentiation Factor-15, Galectin-3, E-Cadherin in Benign Prostatic Hyperplasia Patients with Perceived Stress Scale-10 (PSS-10) of ≤13 and >13

Parameters	BPH Patients with PSS ≤ 13 (n = 24)	BPH Patients with PSS > 13 (n = 60)	*P*
Age (in years)	62 ± 7.9	64 ± 8.6	.262
Disease duration^a^ (in years)	4 (1.0-7.5)	2 (1-5)	.198
BMI (kg/m^2^)^b^	24.1 ± 3.3	23.9 ± 3.5	.842
Prostate size (mL)^a^	26.50 (21.25-40.0)	34.50 (22.25-49.75)	.078
Nocturia (no. of times)^a^	3 (2-4)	4 (3-4)	.020
PSA (ng/mL)^a^	1.08 (0.52-1.65)	1.34 (0.53-2.47)	.422
IPSS^b^	11 ± 3	16 ± 4	<.001
Quality of life^a^	3 (2-4)	5 (3-5)	<.001
GDF-15 (pg/mL)^a^	1995.83 (1653.55-2797.41)	1510.00 (1032.06-2351.88)	.036
Galectin-3 (ng/mL)^b^	2.47 ± 0.66	2.75 ± 0.88	.165
E-cadherin (ng/mL)^a^	17.26 (4.82-35.70)	24.63 (9.20-63.22)	.163

*P-*value < .005 was considered statistically significant after adjusting for multiple comparisons using Bonferroni correction.

BMI, body mass index; BPH, benign prostatic hyperplasia; GDF-15, growth differentiation factor-15; IPSS, International Prostate Symptom Score; PSA, prostate-specific antigen; PSS, Perceived Stress Scale.

^a^Results are represented and median (range).

^b^Results are represented as mean ± SD.

**Table 3.** Clinical Characteristics, Growth Differentiation Factor-15, Galectin-3, HNE, E-Cadherin in Benign Prostatic Hyperplasia Patients with Quality of Life Score of ≤3 and >3

Parameters	BPH Patients with QOL ≤ 3 (n = 32)	BPH Patients with QOL >3 (n = 52)	*P*
Age (in years)^a^	64 (56-67)	66 (60-72)	.113
Disease duration (in years)^a^	2 (1-5)	3 (1-6)	.367
BMI (kg/m^2^)^b^	23.2 ± 3.3	24.4 ± 3.5	.132
Prostate size (mL)^a^	26.5 (22.0-39.2)	37.5 (22-50)	.063
Nocturia (no. of times)^a^	3 (2-4)	4 (3-4.7)	<.001
PSA (ng/mL)^a^	1.03 (0.51-1.77)	1.34 (0.53-2.46)	.347
IPSS^b^	11 ± 3.6	17 ± 4.4	<.001
PSS-10 score^b^	13 ± 4.6	19 ± 5.9	<.001
GDF-15 (pg/mL)^a^	2071.86 (1574.01-2471.37)	1485.74 (918.43-2290.87)	.016
Galectin-3 (ng/mL)^b^	2.68 ± 0.86	2.66 ± 0.82	.892
E-cadherin (ng/mL)^a^	17.71 (7.35 – 39.10)	26.12 (8.75 – 68.84)	.183

*P*-value < .005 was considered statistically significant after adjusting for multiple comparisons using Bonferroni correction.

BMI, body mass index; BPH, benign prostatic hyperplasia; GDF-15, growth differentiation factor-15; IPSS, International Prostate Symptom Score; PSA, prostate-specific antigen; PSS-10, Perceived Stress Scale-10; QOL, quality of life.

^a^Results are represented and median (range).

^b^Results are represented as mean ± SD.

**Table 4.** Correlation of Perceived Stress Scale Score and Growth Differentiation Factor-15 with Biochemical Parameters, International Prostate Symptom Score and Quality of Life Score and Prostate Size in Benign Prostatic Hyperplasia (n = 84)

Parameters	PSS score *r*, *P*	GDF-15 *r*, *P*
Age	0.135, .219	0.178, .109
Duration of disease	−0.031, .776	0.030, .785
Prostate size	0.354, .001	−0.051, .642
PSA	0.065, .555	0.155, .160
IPSS	0.649, <.001	−0.314, .004
Nocturia score	0.294, .007	−0.080, .469
Quality of life score	0.619, <.001	−0.284, .009
PSS score	–	−0.346, .001
GDF-15	−0.346, .001	–
Galectin-3	0.129, .243	0.107, .334
E-cadherin	0.211, .054	−0.041, .709

GDF-15, growth differentiation factor-15; IPSS, International Prostate Symptom Score; PSA, prostate-specific antigen; PSS, Perceived Stress Scale.

**Table 5.** Multivariate Analysis to Predict Prostate Size with Age, Body Mass Index, Duration of Disease, Growth Differentiation Factor-15, E-Cadherin, and Galectin-3 as Covariates

Parameters	*R* ^2^	Beta	*P*	95% CI
Age BMI Duration of disease GDF-15 E-cadherin Galectin-3	0.159	0.272 0.108 0.042 −0.059 0.284 0.015	.015 .328 .700 .597 .013 .898	(0.124-1.138) (−0.616 to 1.819) (−0.988 to 1.463) (−0.005 to 0.003) (0.026-0.213) (−4.701 to 5.352)

BMI, body mass index; BPH, benign prostatic hyperplasia; GDF-15, growth differentiation factor-15.

**Table 6.** Multivariate Analysis to Predict Quality of Life with Age, Body Mass Index, Duration of Disease, Growth Differentiation Factor-15, E-Cadherin, and Galectin-3 as Covariates

Parameters	*R* ^2^	Beta	*P*	95% CI
Age BMI Duration of disease GDF-15 E-cadherin Galectin-3	0.175	0.275 0.190 0.053 −0.264 0.246 −0.172	.014 .085 .623 .018 .029 .134	(0.009-0.078) (−0.010 to 0.156) (−0.988 to 1.463) (−0.005 to 0.003) (0.026-0.213) (−4.701 to 5.352)

BMI, body mass index; BPH, benign prostatic hyperplasia; GDF-15, growth differentiation factor-15.

## Data Availability

The data that support the findings of this study are available on request from the corresponding author.
